# Process of developing a cervical cancer education program for female university students in a Health and Physical Education teacher training course: an action research

**DOI:** 10.1186/s12905-023-02273-8

**Published:** 2023-04-11

**Authors:** Hiroko Yako-Suketomo, Kayoko Katayama, Atsushi Ogihara, Mikiko Asai-Sato

**Affiliations:** 1 Japan Women’s College of Physical Education, 8-19-1 Kita-Karasuyama, Setagaya-Ku, Tokyo, 157-8565 Japan; 2 National Cancer Center, 5-1-1 Tsukiji, Chuo-Ku, Tokyo, 104-0045 Japan; 3 Gunma University, 4-2 Aramaki-Cho, Maebashi, Gunma 371-8510 Japan; 4 Kanagawa Cancer Center Research Institute, 2-3-2 Nakao, Asahi-Ku, Yokohama, Kanagawa 241-8515 Japan; 5 Waseda University, 2-579-15 Mikajima, Tokorozawa, Saitama 359-1192 Japan; 6 Department of Obstetrics and Gynecology, Nihon University, 30-1 Oyaguchikamimachi, Itabashi-Ku, Tokyo, 173-0032 Japan

**Keywords:** Cervical cancer education, Health and Physical Education teacher training, Female university students, Action research

## Abstract

**Background:**

The purpose of this study was to develop a cervical cancer education program for students and evaluate the process for female students of an HPE teacher education university who were training to become Japanese Health and Physical Education teachers.

**Methods:**

This study used Action Research (AR) methodology. In developing program, we analyzed the description of the teaching material development process, the lectures, and the students’ report contents, which was the main activity in the program development. Thirty five third- and fourth-year students majoring in health promotion at a Health and Physical Education teacher education university, which trains Health and Physical Education teachers in Tokyo, Japan, participated in this study.

**Results:**

After a review of the prototype version of the cervical cancer education material, six out of nine reviewers determined that it can be published. In the revised cervical cancer education materials, messages from students, university lecturers, and gynecologists have been added as a column in the section on 'how to prevent cervical cancer. Analysis of the contents of the texts (16,792 characters in total) of 35 student reports resulted in the generation of 51 codes, 3 categories, and 15 subcategories.

**Conclusions:**

This study reflects the intentions of female university students to contribute their knowledge to the development of educational materials on cervical cancer, which, alongside the lectures, have deepened the knowledge and awareness of cervical cancer. Based on this, the teaching material development process, lectures by experts, and students’ mindset after learning about cervical cancer is reported in this study. There is a need for more educational programs on cervical cancer that are implemented through the education of female university students.

## Introduction

Cancer accounts for a high percentage of disease burdens in the world [1], especially in Japan where one out of every two nationals are diagnosed with cancer [2]. After enacting the Cancer Control Act in 2006, the government formulated a National Cancer Control Plan in 2007, 2012, and 2017, and promoted cancer control based on consecutive five-year plans. The second and third Cancer Control Plans-2012 and 2017-focused on cancer education and raising public awareness [3, 4]. The 2017–2018 revision of the national curriculum for middle and high school students [5-8] specified that cancer is taught in Health and Physical Education courses. It has been found that primary, secondary, and tertiary prevention measures are mainly studied for lung, stomach, and colorectal cancers. In addition, nationwide surveys have revealed that school children’s awareness of cancer risk factors and their intention to undergo cancer screening is low [9, 10]. The effectiveness of an education model that uses external instructors such as cancer survivors and medical personnel has been evaluated [11]. It is believed that enabling teachers to teach while utilizing outside instructors is effective in deepening children's cancer prevention knowledge and proper understanding of cancer patients [11].

Health and Physical Education is a compulsory subjects in schools throughout Japan. According to the Japanese curriculum, the relationship between cancer and smoking is taught in health classes in the 5th and 6th grades of elementary school (ages 11–12). In elementary schools in Japan, there is a homeroom teacher system in which the teacher teaches all subjects in principle. In junior high and high schools, on the other hand, each subject is taught by a specialized teacher, and health classes that include cancer content are taught by a Health and Physical Education teacher. Therefore, although the effectiveness of using external instructors has been shown [11], the main nurturers in cancer education are homeroom teachers in elementary schools and Health and Physical Education teachers in junior and senior high schools. However, it has also been reported that teachers lack the requisite knowledge of cancer and are therefore reluctant to teach it [12]. The disadvantage of this is that the introduction of outside instructors may reduce teachers’ teaching ability. For teachers to acquire appropriate knowledge of cancer and give guidance with confidence, it is necessary to develop educational programs that respond to this need in teacher training courses, as well as training for in-service teachers.

A common theme in elementary, junior high, and senior high school cancer education is that primary and secondary prevention measures cancer prevention programs, and cancer screening, form the core of the learning content. Regarding secondary prevention, in particular, five sites: the lungs, stomach, large intestine, breast, and cervix, have been specified in the Health Promotion Act, enacted in Japan in 2000, as sites most prone to cancer in Japan [13]. Except for the cervix, four sites are recommended to be consulted about when an individual is in his or her 40 s and 50 s, while cervical checks are advisable at the age of 20 and above. As the recommended age group to start cervical screening includes female university students during their teacher training course, cervical cancer would be an appropriate teaching material model for university female students as part of their learning.

The main measures against cervical cancer are human papillomavirus (HPV) vaccination and the development of a cancer screening program. In Japan, the prevalence of cervical cancer is increasing for women in the 20–40 age group [14]. Under these circumstances, in 2013, the health hazards caused by the side effects of the vaccine were highlighted in the media, and the Japanese government refrained from actively recommending HPV vaccination [15-17]. The consequence of this was that schools in regions where students had previously been provided with cancer education in line with government guidelines had difficulty to teach on HPV vaccination for cancer prevention. In other words, this showed that scientific evidence-based cancer education in Japan is currently very limited due to such political background. In addition, in Japan, the general adult population, including teachers, has limited knowledge of cancer [18] and the cancer screening uptake rate is low at around 50% [14], suggesting that teachers themselves lack experience surrounding cancer [12]. Therefore, it is important for teachers to improve their health literacy, the personal knowledge and competencies that accumulate through daily activities, social interactions, and across generations [19], and to train teachers who are confident in their knowledge of cancer. In the Japanese curriculum, the content curriculum is structured with one goal in mind: to help children acquire health literacy. Cervical cancer is not included in the curriculum itself, but it is indicated that 'cancer' will be covered. Although it is left to the discretion of each school to decide what cancer to cover, it makes sense to use cervical cancer, which has a well-established preventive effect, as a teaching tool and is useful for school health education.

This study aimed to develop a cervical cancer education program for female students of an HPE teacher education university who were training to become teachers of Health and Physical Education in Japan and who have just reached the age for cervical cancer screening cancer screenings and to clarify the process in qualitative descriptive terms.

## Methods

### Design and setting

The research questions for this study were threefold: First, what are the views of different groups of people on the content of the educational materials used to teach cervical cancer to university students in teacher training programs? Second, what content was included in the material and how was it taught? Third, what were the reactions of the university students who received the education? This study used the Action Research (AR) method [20]. AR is called ‘participatory research’ in which researchers enter a site and those on-site also participate. Together with the participants on-site, they promote democratic activities that include academic activities and also those that influence and change society [20]. In this study, the emphasis is not merely on providing cervical cancer education to the college students who are the research subjects, but also on fostering their willingness to take the initiative. The cervical cancer education program developed in this study adopted the AR methodology because students are both recipients and collaborators in the development of the program.

In AR, it is important to visualize and evaluate the activities performed step by step [20]. As such, in this study, the main research methods included the description of the teaching materials development process, the lectures, and the analysis of the report contents for process evaluation. These were the main activities in the program’s development.

The review and revision of the teaching material, which is the input part of the program in this study, was conducted with the involvement of a diverse group of participants—including 12 editorial board members, 9 reviewers, and a total of 35 students. The AR organized those involved in first-, second, and third-person and focuses on engaging others through areas of mutual interest [21]. In this study, the main instructor is distinguished as a university instructor (first person), a student as a recipient of the program (second person), and an expert who closely examines appropriate educational content (third person). Until now, health education in Japan has been based on the teaching guidelines established by the government (Ministry of Education, Culture, Sports, Science, and Technology), and was guided by teachers. However, when specialized content such as cancer education is included, there is a restriction as only university teachers can teach it in the teacher training course. Thus, this study showed the process of developing an educational program while seeking the knowledge of experts and evaluating students’ responses to handling new learning content.

### Development of educational materials on cervical cancer for university students

The effect of dissemination of cancer knowledge using printed materials has been reported in Japan, mainly in school education [22]. Although not cancer, the effectiveness of printed materials for health education focusing on hypertension management have been reported from occupational fields [23]. A study that distributed health education materials to employees reported that women tend to read printed materials more than men [24]. Since this study aims to develop educational materials from the perspective of university students, we created a twelve-member editorial committee that included five female and two male university students. To ensure the accuracy of medical knowledge and educational considerations, such as cervical cancer, the remaining members comprised two obstetricians and gynecologists specializing in gynecological oncology, one research specialist in epidemiology, and two university faculty members specializing in health promotion and public health, respectively. Based on the teaching materials compiled by the editorial committee, a prototype teaching material for cervical cancer for university students was created. After that, a review was conducted by nine specialists—two gynecological oncologists, two adult women, one adult man, two university faculty members, two female university students, and 1 cancer survivor—followed by the revision and eventual creation of the teaching materials to ensure that the materials are both accurate in their expertise and easy to read for the average student. For the review, a review sheet consisting of the following items was prepared and individually mailed or emailed in January 2017 along with a prototype version of the paper-based teaching materials, and responses were received by February. Regarding the review items, four options (good, need some improvement, need improvement, and could not be judged) were used to check the scientific rationale, comprehensibility, appropriateness of the content, as well as the propriety of publication. After the first edition of the review in March 2017, the material was updated with new content in February 2018 and February 2019, with the final edition published in March 2019.

### Conducting cervical cancer lectures

**Fig. 1:**
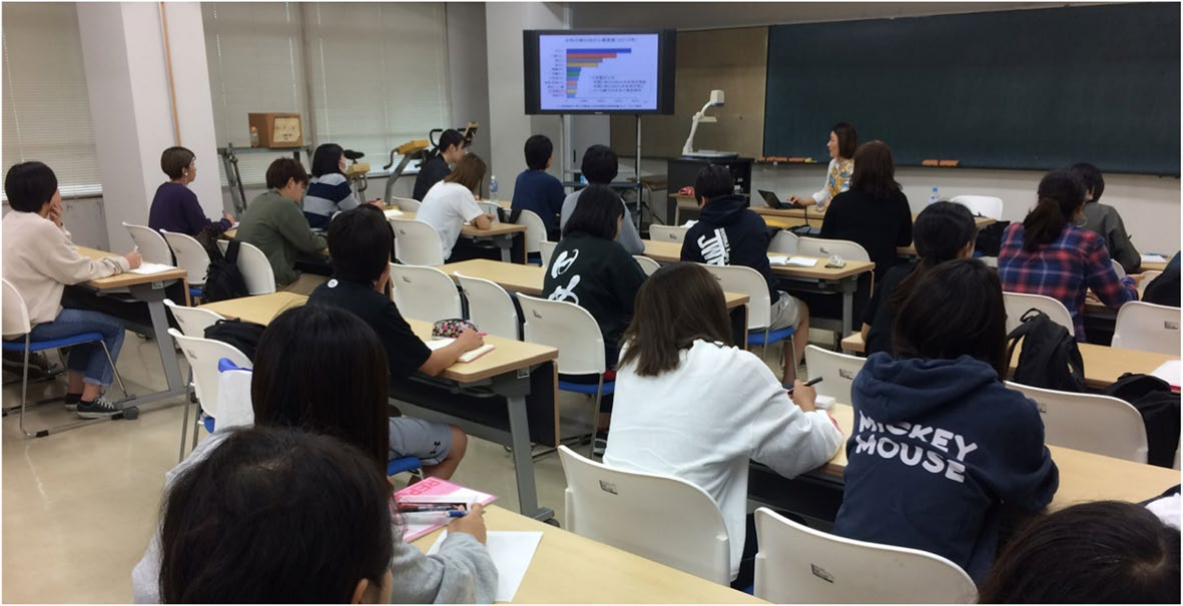
Lecture on cervical cancer

In educational interventions on pregnancy and drinking for female university students in Japan, it has been reported that the dissemination of printed materials along with individual lectures is more effective than disseminating printed materials only [25]. Based on these findings, a total of 35 students in the third and fourth year (20–22 years old) majoring in health promotion at a Women’s Sports and Science University that trains Health and Physical Education teachers in Tokyo were recruited in June 2017 (9 of 14 4th graders and 11 of 15 3rd graders) and October 2018 (11 of 15 fourth graders and 4 of 14 third graders) as participants. In both 2017 and 2018, the first author's seminar recruited students to participate in the study. Eleven students belonging to one academic year participated for two consecutive years. An obstetrician and a gynecologist specializing in gynecological oncology each gave one-hour-long lectures on cervical cancer in 2017 and 2018, respectively (Fig. 1). The lectures conformed to the content of the educational material and covered the epidemiology of cervical cancer, mechanisms of occurrence, risk factors, the effectiveness of the HPV vaccine, and methods of cancer screening. All participants in the lectures were given modified versions of the prototype cervical cancer teaching materials described earlier and were instructed to prepare and review them as part of the class. Furthermore, after completing the review, the students were given the task of preparing a report that included their impressions of the lectures and their aspirations for future learning to capture the transformation of the university students who were the participants in the study.

### Analysis of the content of the reports

After the lecture, the attendance report submitted within one week was converted into text data, and the description was abstracted and coded. After that, the codes were classified by theme using content analysis, and categories were generated which were further classified as subcategories. This classification operation was first performed by the lead author and then modified after consultation with the co-authors until a consensus was reached among all.

## Results

Table 1 shows the results of the review of the prototype version of the teaching material for cervical cancer. Of the nine reviewers, six approved its publication. The remaining three, two adult women and an obstetrician-gynecologist, were undecided about the propriety of publication. The obstetrician-gynecologist decided that medical accuracy should be emphasized, including reference to 'the cytodiagnostic and histological nomenclatures'. Conversely, the two adult women included the implication that they could not make the decision themselves because of the specialized content involved. Of the three viewpoints, the scientific rationale and propriety could not be determined except by obstetricians and gynecologists. All nine reviewers judged it for clarity, one judged that some improvement was required, and the others judged that it was good.

**Table 1:** Prototype version review results

Reviewer	Type	Evaluation results*			Propriety of publication	Comment
		Scientific rational	Ease of understanding	Propriety		
A	Gynecologist (gynecologic oncology	○	○	○	Yes	*Check the color and size of the characters that must be emphasized*
B	Gynecologist (gynecologic oncology	△	△	△	-	*Is there evidence that consuming vegetables and fruits reduces the risk of developing cancer? In recent years, it has become a problem for cancer patients to use extreme diets without evidence, and this statement is of concern. The same goes for the statement that a balanced diet can prevent many cancers* *Is it necessary to explain not only the countermeasure type of screening but also the optional types?* *In recent years, the disadvantages of screening (both in the case of false positives and overdiagnosis/treatment) have also become a problem. In western countries, breast cancer screening and cervical cancer screening are rather ‘I will not do them’ among young individuals* *It is better to mention the disadvantages of screening. At least, I do not agree with nuance statements such as ‘Let's undergo a breast cancer screening even when young’. I am not eligible for screening, however, I think it is good to have awareness of it for the future* *It is very interesting and useful, a distinction can be made between the scientific description before and after. Therefore, it is good to mention a word such as ‘advice from a survivor’ and ‘advice from a senior’* *The item of ‘Bethesda classification’ in the Table is a cytodiagnostic classification, however, the cytodiagnostic and histological nomenclatures are mixed. I think that the citation of Vink *et al*. is only for an ‘expected course’, so it is better to write it accurately* *In the ‘rules for handling cervical cancer’, condyloma acuminatum is the official nomenclature*
C	University lecturer (public health)	○	○	○	Yes	*I thought the content was appropriate* *I think it can be evaluated highly as the examination is devised such that it can be imagined specifically* *However, some places were difficult to read due to the breaks in the text and the centering* *Currently, it is A4 size, but I think that B5 would be fine as it is easier to carry around*
D	University lecturer (Health communication)	○	○	○	Yes	*I thought it would be very useful as material from a woman’s perspective (we were curious about women's concerns, however, we could not ask them about this). Moreover, the sentences are easy to understand. In addition, the illustrations are also fashionable, and the format of the booklet is easy for young women to access* *I thought that the recommended criteria for cancer screening could be updated to the latest ones. If it was due to concerns about character count and space, the present representation is acceptable* *I think that it is better to consistently use Kanji characters (子宮頸 or子宮頚) for the cervix throughout the text unless the two different character forms have been used intentionally. With all due respect, I have made a list of issues that I was concerned about, including typos, in the attached file*
E	Common adult women	-	○	-	-	*I could read many illustrations without difficulty. Though it is a page about cervical cancer, one gets the impression that consultation for cervical cancer starts suddenly. I felt that it would be better to change the page order. (Right)* *Is it easy to use as teaching material if page numbers are inserted throughout? (please ignore if it will be done in the future)*
F	Common adult women	-	○	-	-	*My impression is that it was clear and easy to read. While the design is clear, spreads and sentences on the last page (what is cancer prevention?) were a bit hard to read because there were many line breaks in the middle of the words. The text itself was easy to understand, so if you change just the line feed, it will be smoother to read* *The cover shows that it is for men and women. However, after reading it, I realized it is a book on cervical cancer. So, I was wondering how much interest men will have in it*
G	Student	-	○	-	Yes	*I think the illustration is a very stylish design for our generation* *I think the points and advice on clothes to wear when going for a medical examination would be very helpful. I think it is important to go out and wear the right clothes* *By illustrating the kind of equipment that is used, I felt less anxious* *I think it would be better to appeal from the point that having a health checkup while healthy is necessary* *I think that highlighting the text of the message that needs to be conveyed will make a visual impact* *I think students' medical examination experience may lead to turning the intention to consult a doctor into reality* *I think it would be easy for university students to add a QR code and other tools to connect to the actual screening information site* *Because the font of the characters is the Mincho style and the volume is large, the desire to read it may be low* *When I showed it to the children at the seminar, their impression of the illustration was very high*
H	Student	-	○	-	Yes	*The gender-specific illness rankings are displayed with illustrations, so that gender-specific cancers can be known, and looked out for* *Because the tools and methods used in the examination are illustrated in detail, it is easier to imagine and so one can firmly prepare one's mind for it. On the other hand, one may feel more resistance due to the visual knowledge of the methods and tools used* *For those who have never undergone a medical check-up, it is very helpful to have advice on how to dress on the day* *From someone's experience, the fact that the person can choose a women's clinic with a female doctor tells me that I can go to see a doctor in the future* *Having heard about the experience of a child of the same age gives me a sense of security about the checkup, and also motivates me to go* *Concerning the documented statement that ‘I have to give up pregnancy and childbirth for treatment’, to most women of the same age, the information on not being able to have children is the most shocking and emotional. As it is also linked to future actions, I think it will be easier to communicate if it is emphasized as much as possible* *The point that (it is said to have some effect) is pointed out after the statement that it is not possible to prevent by wearing a condom, the status of the effectiveness is a bit vague* *Regarding the statement that those who have never had sex will benefit little from screening, as I still have many friends (in their 20 s) to whom that applies, it is easy to think that it is information that is not related to me* *The point is ‘will this kind of knowledge cause behavioral changes?’* *The point that the kanji character for ‘cervical’ in cervical cancer is ‘頚 (neck)’ in this sentence only* *In Q3 of the Q&A section, looking at the ‘80% of women who have had sex* ~ *’, and the numbers, one can immediately sense the familiarity with HPV infection, and that can make one aware of the danger* *The Q & As are on one page, making it simple and easy to understand the content* *I feel that there is a possibility that people who are not interested may not read the content to the end because of the large number of sentences* *The design is stylish and the size of the characters is suitable. The colored highlights and font size are easy to understand and are very readable*
I	Cancer survivor	-	○	○	Yes	*Thank you for the deadline. As you know, I am a complete amateur in the medical world, so please forgive my rude suggestions and just disregard them*

*Evaluation results were provided with the following symbols:  〇: Good, △: Some improvement is required, ×: Needs improvement, -: impossible to Judge

Table 2 shows the contents of the cervical cancer education materials before and after the revision. The prototype version was prepared with the contents of the teaching material divided into four main categories: ‘What type of disease is cervical cancer?’, ‘To prevent cervical cancer’, ‘What if I am told I have cervical cancer?’, and ‘Message from cervical cancer survivors. However, in the revised version, ‘Message from cervical cancer survivors’ was deleted, and messages from students, university lecturers, and gynecologists were added as columns in ‘To prevent cervical cancer’. At the moderate item level, nine items were increased to 10, and at the small item level, seven items were increased to 13.

**Table 2:** Contents of the cervical cancer education materials for university students

Major items	Moderate items	Small items
What is cervical cancer?	Symptoms	
	Causes of cervical cancer	HPV
		Transmission route (sex)
		HPV is transmitted to both men and women
	Japanese cancer (statistics of death and morbidity)	
Early detection of cancer	About HPV vaccine	Column: From university faculty
	Who and how often should I have a cervical cancer screening?	Every woman who is 20 years old or older. Every 2 years
	Cancer screening to save many lives: Cancer screening based on scientific evidence	Cancer screening is for those who are healthy
	How is cervical cancer screening performed? (from a survivor)	Brush reference diagram
		When you go for a medical check-up, wear easy-to-wear clothes!
		Column: Student screening experience
		Column: Gynecologist
	Can most cancers be prevented	12 new ways to prevent cancer
	What is the prevention of 'cancer'?	
What if abnormalities are found in the cervical cancer screening?	What if abnormalities are diagnosed as a result of the detailed inspection?	The symptoms and progression
		What is conization?

Table 3 shows the content analysis results of the students’ reports. The total character count in the reports of the 35 subjects was 16,792 characters. From this, 51 codes were obtained, and 15 sub-categories (‘awareness of vaccines’, ‘awareness of the current state of cancer’, ‘awareness of cancer screening’, ‘feeling that cancer is close’, ‘improvement of awareness on the concept of cancer’, ‘Improvement of the willingness to learn’, ‘gratefulness’, ‘improvement of cancer knowledge’, ‘new learning’, ‘difficulty of the course content’, ‘I want to act for myself’, ‘I want to act for my family and friends’, ‘I want to disseminate knowledge on cancer’, ‘important points of communication’, and ‘using them in the future’) were generated.

**Table 3:** Results of content analysis of the student reports

Category	Subcategory	Code
Awareness improvement (7)	Recognition of vaccine (4)	It would be OK if you hit the vaccine (2)
		Vaccines are scary (9)
		No resistance to vaccination (3)
		Vaccination is better (2)
	Recognition of the current state of cancer (5)	Afraid of getting cancer (3)
		Scary/Sad (5)
		There are cancer patients nearby (2)
		Should be interested in health care (6)
		Anxiety has been resolved (1)
	Recognition of cancer screening (4)	I'm afraid of undergoing a cancer screening (2)
		It is better to have cancer screening (7)
		It is difficult to get a cancer screening (5)
		The cancer screening rate is low (6)
	Sinse that cancer is close by (4)	Close to the cancer center (7)
		Ownership (1)
		Patients with cancer (1)
		Familiar diseases (1)
	Raising awareness of cancer information (2)	Need information about cancer screening (2)
		Need information about cancer (15)
	Improve learning motivation (3)	I want to know more about cancer (6)
		The right knowledge can be obtained (1)
		It was a fulfilling training (1)
	Gratitude (1)	Gratitude (9)
Improvement of knowledge level (3)	Improving cancer knowledge (6)	Cancer screening is effective (3)
		Effectiveness of vaccination (4)
		Actual status of cervical cancer (3)
		Cervical cancer and smoking (6)
		Mechanism of HPV infection (1)
		Cervical cancer (2)
	New learning (5)	I learned something (8)
		Learning itself is a valuable experience (3)
		My views changed (2)
		I pretended to know (1)
		I participated for the first time (1)
	The difficulty of the course content (1)	About my understanding (3)
Preparation for action (5)	I want to act for myself (1)	I want to go for cancer screening (6)
	I want to act for my family and friends (4)	Invite family and friends to go for cancer screening (5)
		I wanted to tell my family and friends, and I did (4)
		I want those in the same generation to know (3)
		Tell those close to you (7)
	I want to spread cancer knowledge (4)	I want to spread cancer knowledge (18)
		I want to use it for enlightenment activity (1)
		Local government's recommendation of the examination (1)
		I want to cooperate with smoking cessation (1)
	Important points to convey (3)	Reliable information (1)
		How to convey the information obtained (1)
		Delivering appropriate information to those who need it (1)
	Utilizing the information in the future (4)	I want to make use of in-seminar activities (3)
		I want to apply what I have learned in the future (1)
		I want to make use of my graduation research (1)
		I want to convey this knowledge as a teacher (1)

The number in () indicates the number of sub-items

## Discussion

### Features of this study

The use of AR methodology and female students of a Sports and Science University who are training to become Japanese Health and Physical Education teachers as subjects, and the fact that each phase of development of the cervical cancer education program process was performed based on their participation shows that the study is original.

Health education requires the devising of teaching methods according to the content and the actual situation of the students [26]. In particular, there are difficulties in dealing with cervical cancer, including the issue of adverse reactions to the HPV vaccine [15-17], which has become the subject of active public debate, in school education settings. Therefore, this study provides appropriate educational material contents (input) and the description (output) of the student reports which can aid the training of Japanese Health and Physical Education teachers who are in a social situation where it is difficult to provide cervical cancer education to help develop instructional planning. This shows how much the content is transformed if the curriculum is structured. AR focuses on collaborative problem-solving aimed at creating new wisdom [27]. In this study, we showed what kind of educational content should be provided (input) and the predictability of how students will be transformed (output). Thus, we provided basic materials that can contribute to improving the reproducibility of future programs.

### Appropriate cervical cancer education content

The contents shown in the prototype version of this study were primary, secondary, and tertiary prevention measures including coexistence with cancer survivors that have been made a column, and are common to Japanese cancer teaching materials [28, 29]. However, in the revised content, 'message from cervical cancer survivors' was deleted and placed as a column intended for 'the prevention of cervical cancer'. The messages from students, university lecturers, and gynecologists are predicated on the aim to transform female university students' behavior towards the prevention of cervical cancer, including acquiring HPV vaccination and undergoing screening and to communicate that men are also person concerned. Cancer education for Japanese students focuses on proper awareness of cancer and proper understanding of cancer survivors. In cancer education for children, it was reported that after cancer prevention classes, the percentage of those who chose ‘people who overused cigarettes and alcohol’ as their image of cancer survivors was significantly higher [11].

There is also the possibility that improving cancer prevention awareness for those who equate a lung cancer patient to a smoker [30], and raising issues of cancer stigma [31], will nurture prejudices against cancer patients. In light of this, this study structured the cervical cancer education program to instigate behavioral changes in female university students, and reflect their intentions to use their knowledge to guide cancer education as teachers in the future. To optimize this program, it may be necessary to devise teaching methods, such as inviting cancer survivors as external instructors.

In the Japanese Health and Physical Education teacher training course, knowledge of cancer can be taught in subjects such as public health and health education. However, from the results of the review of the prototype version of the teaching materials of this study, the gynecologists pointed out that cancer risk factors based on epidemiology studies and the limitations of population-based screening, including public health findings, are questionable. Japanese cancer risk factors are smoking, infection, physical inactivity, and inadequate intake of vegetables and fruits [18]. In addition, effective guidelines for cervical cancer screening have also been developed, and they recommend that women aged 20 years and over get examined every other year [13]. On the other hand, there are limitations to providing generalized evidence on cancer prevention to the specific group of university students who are the subjects of this study, and caution is required.

**Fig. 2:**
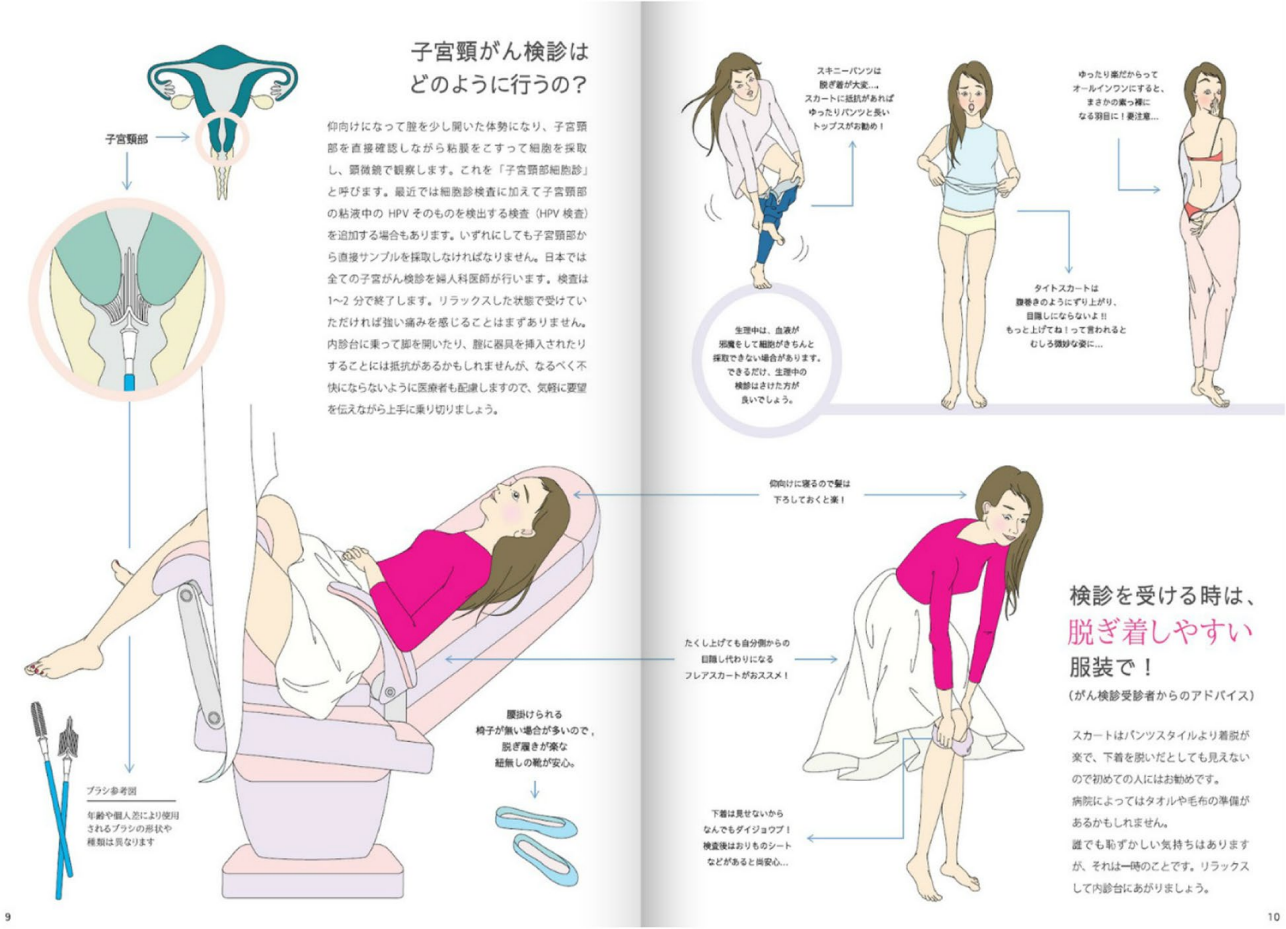
Illustrative example of cervical cancer education materials Partly quoted from Ref. [32]

The content of the educational materials on cervical cancer that was developed in this study is not merely evidence-based knowledge. The revised version of the teaching material content contains illustrations of the brushes used for cervical cancer screening and clothing to put on when going for the screening. It also has a column describing students’ experiences of the medical examination (Fig. 2) [32]. It has been found that women in Japan and the rest of the world, psychologically resist screening with the use of internal examination tables [33, 34]. As such, female university students are vaguely aware of this and may be surprised when faced with the examination table. However, at the time of developing the educational materials, none of the teaching materials explained the specific contents of cervical cancer screening for students. The increase in the number of items related to screening in the revised version of the teaching material content may be the result of those study subjects who have experienced the screening strongly demanding that the content be enhanced.

### The value of this program to the Health and Physical Education teacher training course

Since the United Nations has included education as one of the Sustainable Development Goals (SDGs) [35], it is of great significance to develop human resources who can tackle contemporary health issues in the training of teachers who will be the leaders in this field. In particular, since this study targeted female students, they are expected to take a leadership role in cervical cancer education. In this study, we analyzed the content of the students’ reports and confirmed the structure of their descriptions of statements about the deepening of knowledge and awareness of cervical cancer after the developed program was implemented. In addition, the extraction of the ‘preparation for action’ category confirmed that the college students who participated in the study had the desire to convey the knowledge they had learned to others. Based on the results of the content analysis of the report, the subjects were students training to become teachers of Health and Physical Education, as current students, they would like to convey the acquired knowledge to their immediate families and friends, and as teachers to child students in the future. Such a change, where people are willing to share their knowledge with someone else because of what they have learned, of consciousness that leads to peer education is because women form the majority of health volunteers who disseminate information on health in Japan [36]. These health volunteers have been working well with families and residents while playing a role in maintaining and fostering social capital such as community-based trust and networks [37]. Though there are differences between all 47 prefectures, the pass rate for the teacher recruitment test in Japan in the summer of 2019 was about two to 20 times for junior high school Health and Physical Education, and five to 60 times for high school Health and Physical Education [38]. This shows it is not easy to be hired as a Health and Physical Education teacher. In addition, the percentage of female teachers in Japan is almost the lowest among the Organization for Economic Co-operation and Development countries [39]. As such, even if one obtains a license for teaching Health and Physical Education, the opportunities for using it may be limited. Even under such circumstances, Japanese women have been reported to be able to improve their Quality of Life as full-time housewives [40] and to have a positive effect on others, especially their families. The ripple effect of providing the cervical cancer education program to female university students is significant.

Three categories were extracted in the content analysis of the student report descriptions. Among them, ‘preparation for action’ is a category that was derived from ‘improvement of awareness’ and ‘improvement of knowledge level’. The human mindset and the desire to convey to others when something valuable is learned are explained by social cognitive theory [41]. The Learning Partner Model, which incorporates this, shows the potential for cancer knowledge to spread to others [42, 43]. The concept of the learning pyramid, which shows that active learning has the greatest effect on conveying learning to others, is also shown [44]. Therefore, it is worthwhile to investigate the learning effect and the possibility of its diffusion in the future.

### Limitations and challenges

This study has several limitations. First, it is not clear what parts of the teaching material development and cancer lectures, which are the input of this study, were used in the students; reports. Second, this study used the AR methodology and the subjects are only female university students in Tokyo, Japan. In addition, there may be subject bias. The Hawthorne effect, which feels more promising as a subject of the research per se, causes behavioral changes and eventually leads to favorable results [45]. Third, the most of participants in this study were female university students, and it is unclear from this study whether the results can be applied to male university students. To generalize the usefulness of the teaching materials developed in this study, it is necessary to compare the results with those of the validation conducted at a coeducational general university, which is being conducted in parallel with this study. Forth, the educational materials developed do not include recommendations for HPV vaccination for the prevention of cervical cancer. This was due to public overreaction to adverse reactions to the vaccine in Japan at the time of development. This is because boards of education and others avoided addressing the HPV vaccine issue in cancer education. However, active vaccination recommendations are scheduled to resume after April 2022. From now on, HPV vaccination recommendations can be handled in education.

On the other hand, this study also has some strengths. We used AR in this study. The results of the study showed aspects of Community-Based Participatory Research (CBPR) [46], as there is bidirectional learning and acquisition of knowledge between researchers and the community—in the case of this study, the student population. For this reason, this study has the potential to lead to students being empowered and to knowledge dissemination activities. While paying attention to the interpretation of the results based on the limitations of this study described above, it is necessary to accumulate practices of cervical cancer education programs through education for female university students. Therefore, it is needed to plan to compare the effectiveness of this material and educational program using a literacy scale to compare behavior change (e.g., cancer screening uptake rates) among groups of students with and without peer education.

## Conclusions

In this study, we developed a cervical cancer education program using AR and evaluated the process for female students of an HPE teacher education university who are training to become teachers of Health and Physical Education in Japan and evaluated the process. By so doing, the intentions of the university students were reflected in the developed educational materials for cervical cancer. Moreover, knowledge and awareness of cervical cancer deepened due to the teaching materials, and lectures. A report describing the thoughts of students wanting to convey what they have learned to others was collected. Based on this, certain processes could be visualized. These include the development of teaching materials, lectures by experts, and the mindset of the students after receiving lessons on cervical cancer. In the future, further accumulation of educational programs on cervical cancer that are implemented through the education of female university students is necessary.

## Data Availability

The data generated or analyzed during this study are available from the corresponding author upon reasonable request.

## Abbreviations

AR: Action Research
HPV: Human Papilloma Virus
CBPR: Community-Based Participatory Research
